# Performance of polygenic risk scores in screening, prediction, and risk stratification: secondary analysis of data in the Polygenic Score Catalog

**DOI:** 10.1136/bmjmed-2023-000554

**Published:** 2023-10-17

**Authors:** Aroon D Hingorani, Jasmine Gratton, Chris Finan, A Floriaan Schmidt, Riyaz Patel, Reecha Sofat, Valerie Kuan, Claudia Langenberg, Harry Hemingway, Joan K Morris, Nicholas J Wald

**Affiliations:** 1 Institute of Cardiovascular Science, University College London, London, UK; 2 British Heart Foundation Research Accelerator, University College London, London, UK; 3 National Institute of Health Research Biomedical Research Centre, University College London Hospitals, London, UK; 4 Health Data Research UK, London, UK; 5 University Medical Centre Utrecht, Utrecht, Netherlands; 6 Department of Pharmacology and Therapeutics, University of Liverpool, Liverpool, UK; 7 Precision Healthcare University Research Institute, Queen Mary University of London, London, UK; 8 Computational Medicine, Berlin Institute of Health at Charite Universitatzmedizin, Berlin, Germany; 9 MRC Epidemiology Unit, University of Cambridge, Cambridge, UK; 10 Institute of Health Informatics, University College London, London, UK; 11 Population Health Research Institute, St George's University of London, London, UK

**Keywords:** public health, preventive medicine

## Abstract

**Objective:**

To clarify the performance of polygenic risk scores in population screening, individual risk prediction, and population risk stratification.

**Design:**

Secondary analysis of data in the Polygenic Score Catalog.

**Setting:**

Polygenic Score Catalog, April 2022. Secondary analysis of 3915 performance metric estimates for 926 polygenic risk scores for 310 diseases to generate estimates of performance in population screening, individual risk, and population risk stratification.

**Participants:**

Individuals contributing to the published studies in the Polygenic Score Catalog.

**Main outcome measures:**

Detection rate for a 5% false positive rate (DR_5_) and the population odds of becoming affected given a positive result; individual odds of becoming affected for a person with a particular polygenic score; and odds of becoming affected for groups of individuals in different portions of a polygenic risk score distribution. Coronary artery disease and breast cancer were used as illustrative examples.

**Results:**

For performance in population screening, median DR_5_ for all polygenic risk scores and all diseases studied was 11% (interquartile range 8-18%). Median DR_5_ was 12% (9-19%) for polygenic risk scores for coronary artery disease and 10% (9-12%) for breast cancer. The population odds of becoming affected given a positive results were 1:8 for coronary artery disease and 1:21 for breast cancer, with background 10 year odds of 1:19 and 1:41, respectively, which are typical for these diseases at age 50. For individual risk prediction, the corresponding 10 year odds of becoming affected for individuals aged 50 with a polygenic risk score at the 2.5th, 25th, 75th, and 97.5th centiles were 1:54, 1:29, 1:15, and 1:8 for coronary artery disease and 1:91, 1:56, 1:34, and 1:21 for breast cancer. In terms of population risk stratification, at age 50, the risk of coronary artery disease was divided into five groups, with 10 year odds of 1:41 and 1:11 for the lowest and highest quintile groups, respectively. The 10 year odds was 1:7 for the upper 2.5% of the polygenic risk score distribution for coronary artery disease, a group that contributed 7% of cases. The corresponding estimates for breast cancer were 1:72 and 1:26 for the lowest and highest quintile groups, and 1:19 for the upper 2.5% of the distribution, which contributed 6% of cases.

**Conclusion:**

Polygenic risk scores performed poorly in population screening, individual risk prediction, and population risk stratification. Strong claims about the effect of polygenic risk scores on healthcare seem to be disproportionate to their performance.

**WHAT IS ALREADY KNOWN ON THIS TOPIC**

- Disagreement exists on the performance of polygenic risk scores in screening, prediction, and risk stratification, and therefore their potential value in healthcare

**WHAT THIS STUDY ADDS**

- Calculating informative performance metrics for 926 polygenic risk scores for 310 diseases from the Polygenic Score Catalog indicated poor performance in screening (median detection rate for a 5% false positive rate of 11%), with correspondingly poor performance in individual risk prediction and population risk stratification

**HOW THIS STUDY MIGHT AFFECT RESEARCH, PRACTICE, OR POLICY**

- The wide scope and analytical approach of this study should resolve disagreement on the value of polygenic risk scores and avoid unjustified expectations about their role in preventing disease

## Introduction

A polygenic risk score represents the weighted sum of independent DNA sequence variants present in an individual's genome that are associated with the risk of a particular disease.^1^ The weight assigned to each variant is based on the strength of its disease association, estimated from a genome wide association study. The increasing range and scale of genome wide association studies over the past decade, now spanning more than 2500 diseases or traits,^2^ has proliferated polygenic risk scores, with widespread interest in potential healthcare applications^3^ and attention from policy makers.^4^

Claims have been made that polygenic risk scores generate "substantial" improvements in risk prediction,^5^ will "power a transformative change to healthcare",^6^ and should be made ready to implement in practice.^7 8^ In a move towards clinical implementation, position papers have been published on reporting standards and responsible clinical use from the Clinical Genome Resource (ClinGen) Complex Disease Working Group^9^ and the Polygenic Risk Score Task Force of the International Common Disease Alliance.^10^ Individual consumers and healthcare providers can already access commercial genetic testing and software services based on polygenic scores.^11–13^ A "world first" pilot trial of predictive genetic testing for cardiovascular disease is also underway in participants attending vascular health checks in the NHS,^14^ and genetic risk scores for prediction of disease are central to the aims of the Our Future Health programme aiming to recruit five million UK adults.^15^

These claims, however, are disputed^16–19^ resulting in disagreement on the performance of polygenic risk scores in population screening, individual risk prediction, and population risk stratification, making their role in medicine and public health uncertain. Recently, Lambert and colleagues produced the Polygenic Score Catalog, a comprehensive, regularly updated, open access directory of studies on polygenic scores for quantitative traits (eg, blood pressure) and polygenic risk scores for diseases (eg, breast cancer).^20 21^ The catalogue lists the performance metrics for polygenic risk scores as hazard ratios or odds ratios for an increment of one standard deviation in the score, area under the receiver operating characteristic curve, or the C index.

But these metrics are not directly informative of performance in population screening, individual risk prediction, and population risk stratification. Screening is defined as "the systematic application of a test or enquiry to identify individuals at sufficient risk of a specific disorder to benefit from further investigation or direct preventive action, among persons who have not sought medical attention because of symptoms of that disorder."^22^ Individual risk prediction is the estimation for an individual of the risk of becoming affected in a given time frame, and population risk stratification is the estimation for a population subgroup of the absolute risk (or odds) of becoming affected in a given time frame.

An appropriate performance measure is the odds of becoming affected, which is the positive predictive value expressed as an odds. For example, an odds of 1:9 equates to a risk of one in 10 or 10%. The odds of becoming affected is calculated by multiplying the background odds of developing disease in a specified time frame by the likelihood ratio associated with a positive test (population screening), with a particular polygenic score value (individual risk prediction), or occupancy of a particular polygenic risk score quantile group (population risk stratification).

We mathematically derived the odds of becoming affected in each of these scenarios based on the metrics reported in the Polygenic Score Catalog. We used breast cancer and coronary artery disease as illustrative examples and scrutinised two proposed early clinical uses of polygenic risk scores: to improve on the performance of the established risk factor models in the prediction of coronary artery disease and stroke, and to prioritise mammographic screening at a younger age for the detection of breast cancer.

## Methods

### Reported performance metrics

By April 2022, the Polygenic Score Catalog had curated 13 828 performance metric estimates for 2194 polygenic scores (unique polygenic score codes), for 544 diseases or traits (unique experimental factor ontology identifiers), reported in 303 unique publications. We removed polygenic scores for continuous traits and those with implausible values (167 instances where the hazard ratio or odds ratio for one standard deviation was recorded as <1, two instances where the area under the receiver operating characteristic curve was <0.5, and one instance where the C index was recorded as 632), leaving 3915 performance metric estimates for 926 polygenic risk scores with 310 unique binary outcomes (mainly diseases). The reported performance metrics were odds ratio for one standard deviation in 1216 instances, hazard ratio for one standard deviation in 378, area under the receiver operating characteristic curve in 2077, and C index in 244 instances (online supplemental file 1).

10.1136/bmjmed-2023-000554.supp1 Supplementary data

### Converting reported metrics to useful performance metrics

Polygenic risk scores have a gaussian distribution with the same standard deviation in affected and unaffected groups (online supplemental file 2). We used these properties together with the metrics reported in the Polygenic Score Catalog to mathematically derive measures that are more useful for judging the performance of polygenic risk scores in their intended applications.

10.1136/bmjmed-2023-000554.supp2 Supplementary data

The first step in this calculation is to use the reported metrics to calculate the difference in mean values for polygenic risk scores between affected and unaffected groups. The calculations have been described previously^23–25^ and are explained in detail in online supplemental file 2. We used the calculated difference in mean values to determine the overlap in the distributions of the polygenic risk score between the affected and unaffected groups. This method allows calculation of the detection rate and false positive rate, which are the percentage of people with a polygenic score above a particular cut-off value (positive test) among those who are later affected or remain unaffected by disease, respectively. For simplicity and consistency, we set the polygenic score cut-off value at the 95th centile for the unaffected group (1.645 standard deviation units from the mean). This cut-off value defines a 5% false positive rate, and the corresponding detection rate (DR_5_) is the detection rate for a 5% false positive rate.^23 25^ The work sheets in online supplemental file 1 can be used to enter the odds ratio or hazard ratio for one standard deviation, area under the receiver operating characteristic curve, or the C index and return the DR_5_ value. A risk screening converter that returns the detection rate for any user defined false positive rate value is available at https://www.medicalscreeningsociety.com/rsc.asp.

**Figure 1:**
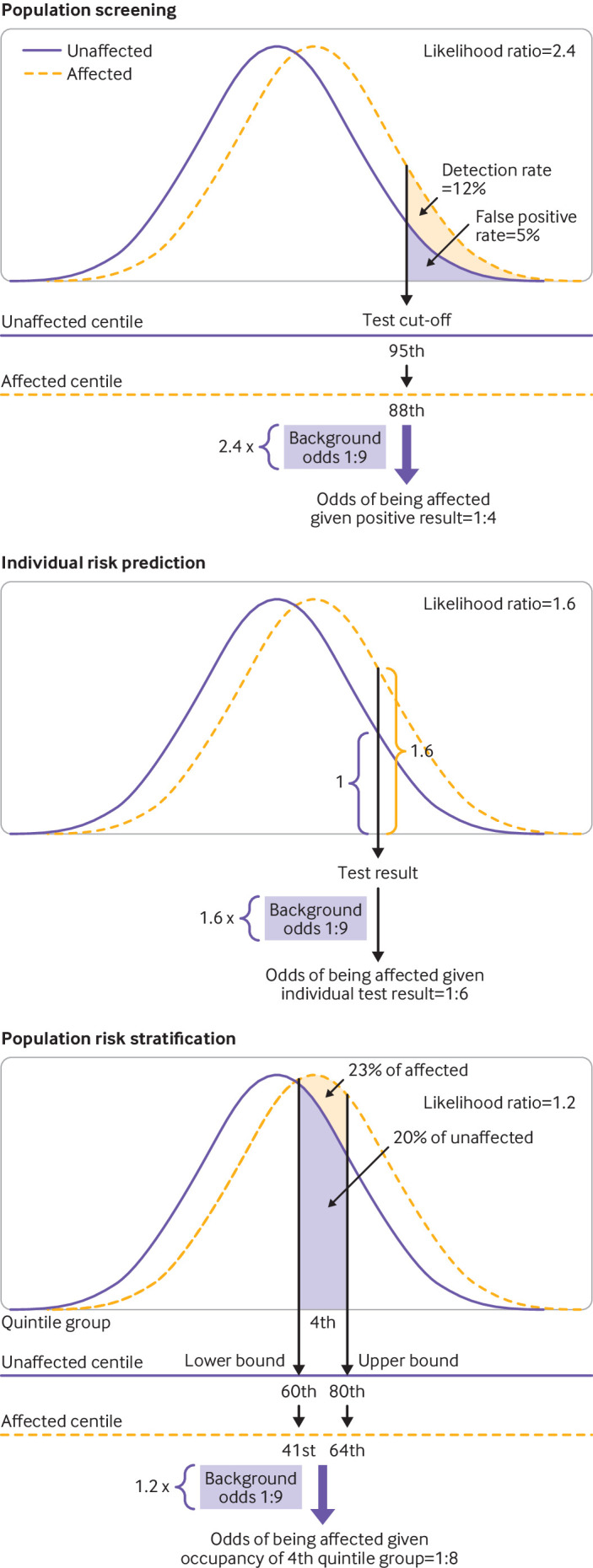
Derivation of metrics useful in assessing performance of polygenic risk scores in population screening, individual risk prediction, and population risk stratification. Difference in mean values for polygenic risk scores between affected and unaffected groups (and standard deviations) allows determination of overlap in polygenic risk score distributions between the two groups. Likelihood ratio in screening is detection rate for a specified false positive rate (5%) and is the ratio of the shaded areas in the top panel. In individual risk prediction, likelihood ratio is the ratio of the heights of the distributions at a specified polygenic risk score (middle panel). In population risk stratification, likelihood ratio is the ratio of areas under the distributions for a specified group of the population (fourth quintile group in bottom panel). Multiplication of the likelihood ratio by the background odds of disease in the population (1:9) allows calculation of the odds of becoming affected for each patient

The likelihood ratio in screening is the ratio of the detection rate/false positive rate. In individual risk prediction, the likelihood ratio is the ratio of the heights of the gaussian distribution curves for affected and unaffected individuals at a particular polygenic risk score centile. In risk stratification, the likelihood ratio is the ratio of areas under the relative frequency distributions for affected and unaffected individuals in each polygenic score quantile (eg, each fifth of the polygenic score distribution; figure 1 and online supplemental file 2). In each case, multiplying the likelihood ratio by the background odds of disease for the whole population gives the corresponding odds of becoming affected for the individual or group of interest. When we discuss a polygenic score centile or quantile, we are referring to the distribution in the unaffected group. When referring to a particular polygenic risk score, we used the Polygenic Score Catalog identifier number.

We reanalysed tabular data taken from two original sources^5 26^ to quantify the extent to which the addition of information on polygenic risk score to data on conventional risk factors improved the prediction of coronary artery disease and stroke. For this analysis, we extracted the reported counts of affected and unaffected individuals above and below the 10 year risk cut-off values recommended in guidelines for starting treatment with statins. We did this analysis separately for the counts reported by the authors for conventional risk factor models, and for the conventional risk factor models with the addition of polygenic risk scores (online supplemental file 1). We used these counts to calculate the detection rate and false positive rate with and without information on the polygenic risk score. We then calculated the number of individuals who need to be genotyped (and a polygenic score calculated) to detect or prevent one additional coronary artery disease event or stroke with the authors own assumptions about the risk reduction from treatment with statins. We refer to this value as the number needed to genotype (table 1). We modelled the use of a breast cancer polygenic risk score to prioritise mammographic screening at age 40 rather than from the currently recommended age of 50.

**Table 1:** Effect of adding polygenic risk score to non-genetic risk factors in prediction of coronary artery disease and stroke

Study	Population screened (cardiovascular events)	Risk assessment	Detection rate (%)	False positive rate (%)	No of patients prescribed statin	Cardiovascular events above cut-off value	Events prevented	Number needed to genotype
Sun et al^26^	100 000 (7997)	Conventional risk factors	60	24	26 722	4783	957	—
		Conventional risk factors+polygenic risk score	61	23	26 445	4870	974	5882
Riveros et al^5^	186 451 (4247)	QRISK3	81	42	79 754	3450	690	—
		QRISK3+polygenic risk score	84	41	78 092	3557	711	8879

Values are based on data from Sun et al^26^ and Riveros-Mckay et al.^5^ Both studies used data from UK Biobank. Sun et al developed a conventional risk factor model and examined the effect of adding polygenic risk scores for coronary artery disease (Polygenic Score Catalog identifier PGS000018) and stroke (PGS000039) on the prediction of subsequent coronary artery disease and stroke events. Sun et al used a 10 year risk cut-off value of 10% for prescribing treatment with statins. Riveros-McKay et al^5^ modelled screening performance in 186 451 participants based on the cardiovascular risk score, QRISK3, also with a 10% risk cut-off value for prescription of statins. Data on events reported by Riveros-Mackay et al^5^ were for coronary artery disease only rather than coronary artery disease and stroke. Calculations assume that all those exceeding the specified risk cut-off value receive a statin, 100% adherence, and that statin treatment produces a 20% reduction in relative risk. Counts were used to calculate number needed to genotype: number of individuals that need to be genotyped (and have a polygenic risk score calculated) to detect or prevent one additional cardiovascular event (see text).

### Patient and public involvement

Patients and the public were not involved in the design, or conduct, or reporting of this research. We plan to work with patients and the public to disseminate the findings through the patient and public representative groups of the Multimorbidity Mechanisms and Therapeutics Research Collaborative and the UCL Hospitals NIHR Biomedical Research Centre.

## Results

### Performance of polygenic risk scores in screening

**Figure 2:**
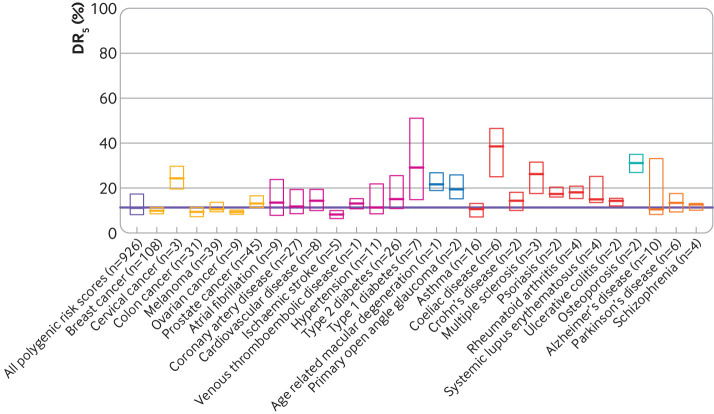
Performance in screening estimated for polygenic risk scores included in the Polygenic Score Catalog from April 2022. Limits of each box represent interquartile range and horizontal line within each box is estimated detection rate for a 5% false positive rate (DR_5_) based on performance metrics reported for corresponding polygenic risk scores. Selected diseases are colour coded into categories cancers, cardiometabolic conditions, ocular diseases, allergic or autoimmune diseases, bone disease, and neuropsychiatric diseases. Horizontal line is estimated median DR_5_ value based on performance metrics for all 926 polygenic risk scores and all diseases studied in the Polygenic Score Catalog

For all diseases studied, median DR_5_ based on all polygenic risk scores was 11% (interquartile range 8-18%); that is, 89% (82-92%) of patients were missed. Median DR_5_ values for polygenic risk scores whose performance was reported with odds ratio or hazard ratio for one standard deviation were 9% (6-12%) and 8% (7-10%), respectively. For polygenic risk score performance reported based on area under the receiver operating characteristic curve or the C index, median DR_5_ values were 14% (10-22%) and 19% (13-25%), respectively. Figure 2 shows median DR_5_ values for polygenic risk scores for 28 common diseases, including coronary artery disease and breast cancer.

#### Coronary artery disease

**Figure 3:**
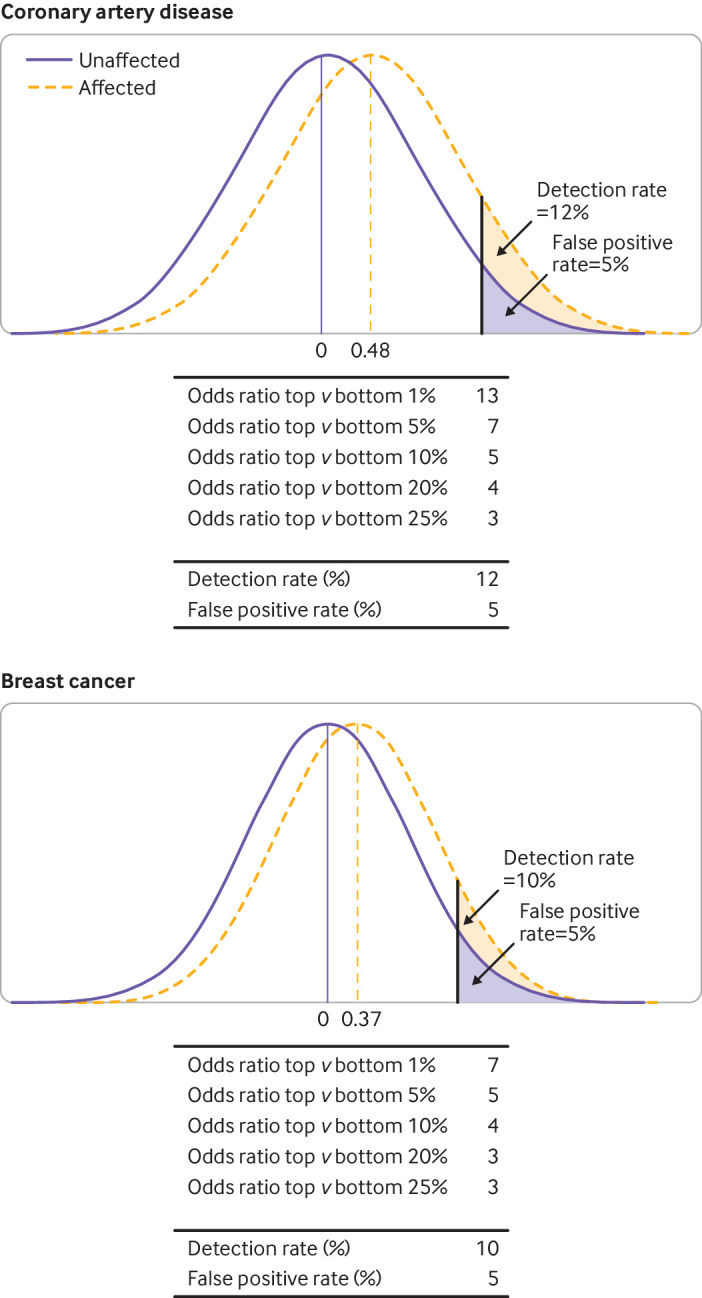
Relative polygenic risk score distributions among those later affected or not by coronary artery disease and breast cancer. Mean value of polygenic risk score distribution in those later affected was shifted 0.48 standard deviation units to the right of the mean of the distribution for those who remained unaffected by coronary artery disease, and 0.37 standard deviation units to the right for breast cancer. Also shown are corresponding values for detection rate for a 5% false positive rate (DR_5_) and for odds ratios (rounded to the nearest whole number) for comparisons of top and bottom 1%, 5%, 10%, 20%, and 25% of unaffected polygenic risk score distribution

Median DR_5_ from performance metrics for 27 polygenic risk scores for coronary artery disease was 12% (interquartile range 9-20%; 88% of patients missed) (figure 3), corresponding to a likelihood ratio of 2.4. Applied at age 50, with a background 10 year risk of coronary artery disease of 5% (odds 1:19), the odds of becoming affected given a positive result were 2.4:19 or about 1:8; that is, false positive results outnumbered true positive results by about eight to one. Reducing the cut-off value to reduce the false positive rate to 1% reduced the detection rate to 3%, with 97% of patients missed. Retaining a 5% false positive rate but applying the test in a population with a risk of coronary artery disease of one in 56 (about 2%), over the same period (background odds 1:55; eg, at about age 40 years of age), gave an odds of becoming affected given a positive result of 1:23, with false positive results outnumbering true positive results by just over 20 to one.

#### Breast cancer

Median DR_5_ from performance metrics reported for 108 polygenic risk scores for breast cancer was 10% (interquartile range 9-12%; 90% of patients missed) (figure 3), corresponding to a likelihood ratio of 2. Applied at age 50, with a background 10 year risk of breast cancer of about 2.5% (odds 1:41), the odds of becoming affected given a positive result were 1:21. Applying the polygenic risk score as a test at age 40, when the background 10 year odds was 1:64, gave an odds of becoming affected given a positive result of 1:32, with false positive results outnumbering true positive results by just over 30 to one.

### Performance of polygenic risk scores in individual risk prediction

The overlap in distributions of polygenic scores derived from the metrics in the Polygenic Score Catalog allowed calculation of the likelihood ratio for an individual which, together with the background odds of the disorder for the population, can be used to calculate the odds of becoming affected for that individual (online supplemental file 2).^25^

#### Coronary artery disease

**Figure 4:**
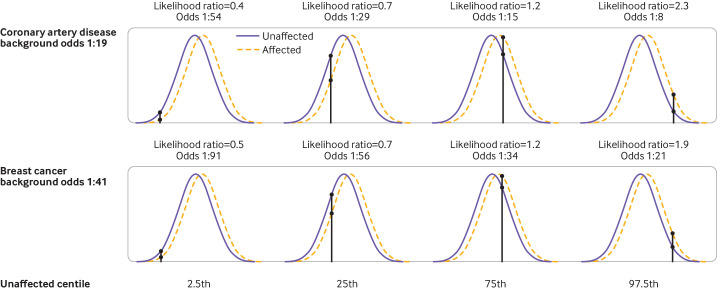
Likelihood ratios and 10 year odds of coronary artery disease and breast cancer for people aged 50 with a polygenic risk score result corresponding to 2.5th, 25th, 75th, and 97.5th centiles of the corresponding distribution

The odds of developing coronary artery disease in the next 10 years were 1:54, 1: 29, 1:15, and 1:8 with a polygenic risk score at the 2.5th, 25th, 75th, and 97.5th centiles, respectively, at age 50 (when the background odds was 1:19) (figure 4), and 1:157, 1:85, 1:45, and 1:24, respectively, at age 40 (when the background odds was 1:55).

#### Breast cancer

The average 10 year odds of breast cancer was 1:41 for a woman aged 50 and 1:64 for a woman aged 40. The corresponding odds of being affected were 1:91, 1:56, 1:34, and 1:21, respectively, at age 50 (figure 4), and 1:142, 1:88, 1:53, and 1:33 at age 40, for a woman with a polygenic risk score at the 2.5th, 25th, 75th, and 97.5th centiles, respectively.

### Performance of polygenic risk scores in risk stratification

#### Coronary artery disease

**Figure 5:**
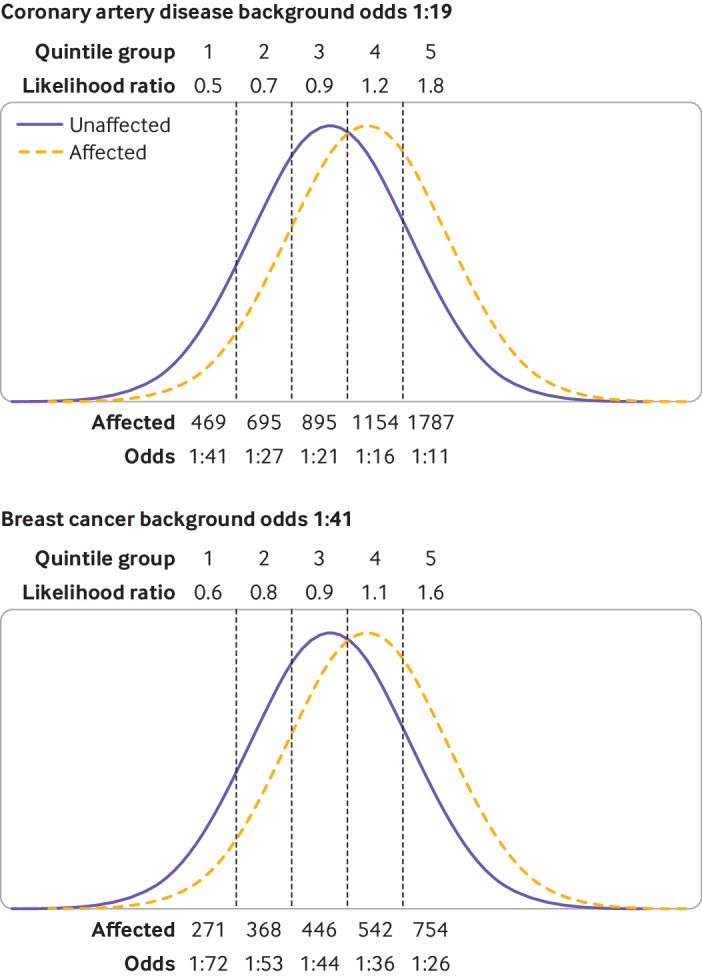
Likelihood ratios, odds, and number of affected and unaffected individuals for each quintile group in a hypothetical population of 100 000 individuals with a background 10 year odds of coronary artery disease of 1:19, and women with a 10 year odds of breast cancer of 1:41

**Figure 6:**
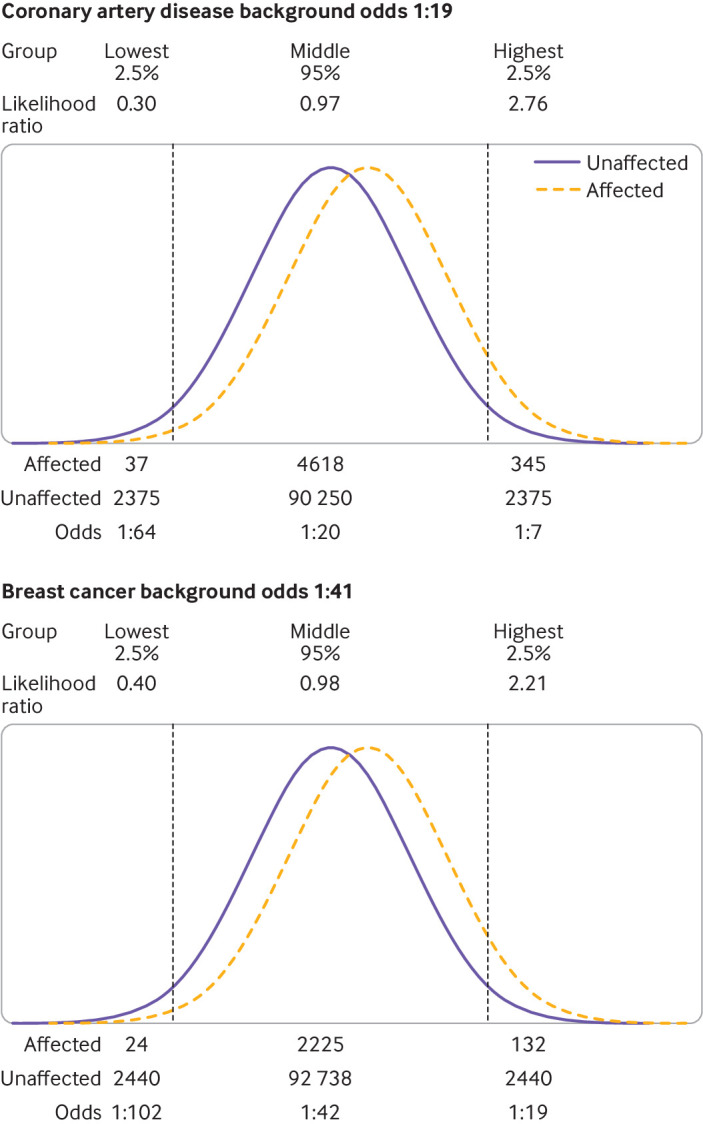
Likelihood ratios and 10 year odds of coronary artery disease and breast cancer for people aged 50 comparing highest and lowest 2.5% of the unaffected polygenic risk score distributions

Figure 5 shows the overlapping distributions for affected and unaffected individuals applied to a hypothetical cohort of 100 000 men aged 50 years grouped into polygenic risk score quintile groups. The 10 year odds of coronary artery disease were reduced from the average of 1:19 for all men to 1:41 for those in the lowest quintile group, and increased to 1:11 in the highest quintile group. Figure 6 focuses on those at the highest risk, and the 10 year odds were 1:7 for the upper 2.5% of the polygenic risk score distribution for coronary artery disease, but this group contributed only 7% of patients.

#### Breast cancer

We applied the same approach to a hypothetical cohort of women aged 50 with a background 10 year odds of breast cancer of 1:41 (figure 5). The odds reduced by about half to 1:72 for those in the lowest quintile group and almost doubled to about 1:26 for those in the highest quintile group of the polygenic risk score distribution. For the highest 2.5% of the polygenic risk score distribution (figure 6), the odds were increased to 1:19, but the latter group only accounted for 6% of patients with breast cancer.

### Polygenic risk scores for screening along with conventional risk factors or tests

#### Coronary artery disease

Adding polygenic risk scores to conventional risk factors (eg, blood pressure and low density lipoprotein cholesterol) has been proposed to usefully improve coronary artery disease and stroke screening to indicate who should be prescribed a statin for primary prevention. Table 1 shows the results from Sun et al,^26^ applied in a hypothetical cohort of 100 000 individuals aged 40 with a risk factor profile representative of the English population and a background 10 year risk of coronary artery disease and stroke of 8%. A conventional, multi-risk factor model incorporating age and a 10 year risk cut-off value of 10%, detected 60% of those later affected by coronary artery disease or stroke at a false positive rate of 24% (DR_24_=60%). The addition of polygenic risk scores for coronary artery disease and stroke to the model (Polygenic Score Catalog identifiers PGS000018 and PGS000039, respectively) detected 61% of those affected with a false positive rate of 23% (DR_23_=61%). Assuming a 10 year risk cut-off value of 10% for prescribing statins,^27^ 100% adherence, and adopting the assumption of Sun et al that statins reduce the risk of coronary artery disease and stroke by 20%,^26^ 974 events would be prevented with a model based on conventional risk factors and polygenic risk scores compared with 957 with a conventional risk factor model and no genetic information, a gain of 17 patients prevented (table 1).

This method gives a number needed to genotype to prevent one additional event of 5882. Sun et al also estimated that 1029 coronary artery disease and stroke events would be prevented with a hybrid model, when conventional assessment of risk factors is followed by polygenic risk scores only for those with an intermediate (5-10%) 10 year risk. Replacing this more complicated model, however, with one where the whole cohort receives statins would prevent 1600 cardiovascular events based on the same assumptions (online supplemental file 1). Because age is a major determinant of the risk of coronary artery disease and stroke, age alone performs about as well as multiple risk factor models that include age.^28^ Based on the rarity of coronary artery disease and stroke events at age <50, an age cut-off of 50 instead of 40 would prevent almost as many events but with fewer false positive results.^29^

Similar results were obtained with Riveros-McKay et al's data.^5^ These authors also investigated the extent to which the addition of a polygenic risk score to conventional risk factors improved the identification of UK Biobank participants eligible to receive statins because their 10 year risk of coronary artery disease and stroke exceeded the cut-off values used in UK or US primary prevention guidelines. Deriving the appropriate metrics from their data (table 1 and online supplemental file 1) clarifies the effect of adding information from a polygenic risk score for coronary artery disease. With a 10 year risk cut-off value of 10% for starting statins, the cardiovascular risk score, QRISK3 model, based on conventional risk factors including age, detected 81% of patients at a false positive rate of 42% (DR_42_=81%). The addition of a polygenic risk score to the model detected 84% of patients for a false positive rate of 41% (DR_41_=84%). Based on the authors' assumption that statins reduce coronary artery disease and stroke events by 20%, 711 events would be prevented with a model based on conventional risk factors and polygenic risk scores compared with 690 with a conventional risk factor model and no genetic information, a gain of 21 patients prevented. This calculation gives a number needed to genotype to prevent one additional event based on this study of 8879.

#### Breast cancer

**Figure 7:**
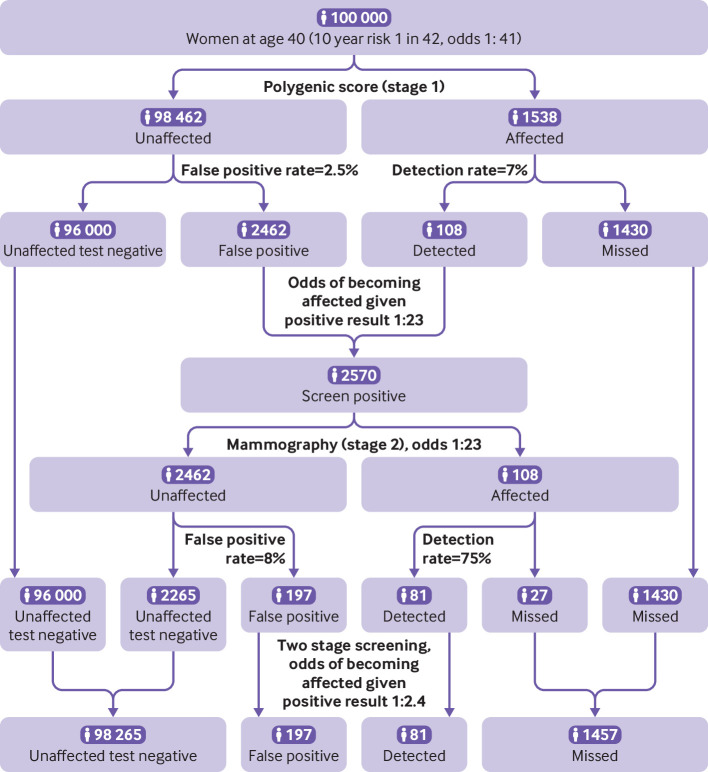
Estimated number of patients with breast cancer detected and missed, number of false positive results, and number of additional mammograms for a two stage screening test with a polygenic risk score (Polygenic Score Catalog identifier PGS000004) with a cut-off value at the unaffected 97.5th centile. Estimates are based on a hypothetical cohort of 100 000 women aged 40 with a background 10 year odds of breast cancer of 1:41. Performance of mammography in the detection of breast cancer uses estimates from the literature^31^

Using polygenic risk scores to prioritise the use of established screening tests for cancer has also been proposed.^3^ One suggestion is that younger women should undergo mammographic screening if their risk of breast cancer, determined with a polygenic risk score, exceeds that of an average woman aged 50, the age when mammography is offered to all women. Figure 6 shows that women aged 40 at or above the unaffected 97.5th centile of a breast cancer polygenic risk score distribution have an odds of breast cancer of 1:19, higher than the average 10 year odds at age 50 of 1:41.^30^ Figure 7 shows that by using the breast cancer polygenic risk score (Polygenic Score Catalog identifier PGS000004) as a stage 1 screen in 100 000 women aged 40, applying the unaffected 97.5th centile as a cut-off value would result in 2570 women with a high risk polygenic score being offered mammography, of whom 108 would be affected and 2462 unaffected (odds of becoming affected given a positive result 1:23). Assuming 100% uptake and a DR_8_ value of 75%,^31^ mammography would then correctly identify 81 of the 108 affected individuals but miss 27 patients with breast cancer. However, 1430 patients with breast cancer (over 10 times as many) are estimated among the 97 430 women aged 40 with a polygenic risk score below the unaffected 97.5th centile who would not be offered mammography.

## Discussion

### Principal findings

Our results showed the poor performance of polygenic risk scores in population screening, individual disease prediction, and population risk stratification. This finding is not obvious from the metrics reported in the Polygenic Score Catalog but is clear based on the appropriate metrics used in this study. Our conclusion is consistent with that of others,^16 17 19^ but is insufficiently recognised. The findings are relevant to consumers, patients, doctors, those involved in preventive medicine and public health, as well as funders and policy makers.

Polygenic risk score distributions overlapped substantially for all conditions studied, and this extensive overlap constrained their performance in each of their intended applications, whether used alone or in combination with conventional risk factors or screening tests. For example, achieving a clinically useful performance in population screening, such as an 80% detection rate for a 5% false positive rate (DR_5_=80%) requires an odds ratio for one standard deviation of 12 or higher (compared with the median observed value of 1.31) or an area under the receiver operating characteristic curve of 0.96 (compared with the median observed value of 0.65). Only 11.4% of the area under the curve values in the Polygenic Score Catalog exceeded 0.8, which equates to a DR_5_ of 32%, with most of these resulting from large effect variants at the HLA locus in a few autoimmune diseases (figure 1 and online supplemental file 1).

### Study implications

When a risk factor has a monotonic relation with risk of disease,^32^ more instances arise among the majority with near average risk factor values than among the few with more extreme values, termed the prevention paradox.^33 34^ In this respect, polygenic risk scores are similar to some non-genetic risk factors, such as blood pressure and low density lipoprotein cholesterol, which although causal, are poor predictors of coronary artery disease.^16 35^ That the performance of polygenic risk scores in the prediction of coronary artery disease is sometimes compared favourably with that of blood pressure and cholesterol^26^ is to benchmark one poor predictor against another.

Where safe and inexpensive preventive interventions are available (eg, statins and blood pressure lowering drugs for prevention of coronary artery disease and stroke), broadening rather than limiting eligibility for such interventions gives greater public health benefits.^36^ Prevention of coronary artery disease and stroke has been achieved in effect by the progressive lowering of the 10 year risk cut-off value for prescription of statins in primary prevention. The cut-off value was reduced from a 10 year risk of coronary artery disease in the UK in 1997 of 30%,^37^ to 10% for the 10 year risk of coronary artery disease or stroke in the UK from 2016^27^ and to 7.5% in the US from 2019.^38^ The reduction in the risk cut-off value resulted from reduced drug acquisition costs through patent expiry, and by accumulating evidence on long term safety. Eligibility could be extended even further and simplified by using age alone to guide prescription of statins for primary prevention, preventing coronary artery disease and stroke in many more patients.^28^ In contrast, retaining the same 10 year risk cut-off value and adding information on polygenic risk score to conventional risk factor models has a much weaker effect. Based on recently reported data,^5 26^ we showed that several thousand individuals need to be genotyped and a polygenic risk score calculated to prevent one additional vascular event.

Identifying a minority of individuals at very high risk (with genetics or other means) might be justified if a preventive intervention is costly, resource limited, or has substantial harms.^39^ With breast cancer as an example, however, we showed that identifying those at high risk requires testing in all and, apart from missing the many more patients among those at average risk, generates many false positive results. This finding could have substantial downstream resource implications for healthcare systems if, for example, genetic risk stratification was followed by a confirmatory screening test, such as mammography for breast cancer.^40^ In this case, reducing the age cut-off value for mammography for all women without determining their polygenic risk score might be more sensible.

The enthusiasm surrounding polygenic risk scores might have been encouraged by pressure on academia to demonstrate a tangible health effect after decades of research investment in human genomics and by commercial opportunity. Unrealistic expectations have probably been raised by use of uninformative metrics. Publications on polygenic risk scores often illustrate comparisons between mutually exclusive groups (eg, those in opposite ends of a polygenic score distribution).^41^ This finding is relevant in aetiological studies but is not relevant in screening. Figure 3 shows seemingly impressive odds ratios of 13, 7, 5, 4, and 3 for comparisons of the top versus the bottom 1%, 5%, 10%, 20%, and 25%, respectively, of the polygenic risk score distribution for coronary artery disease, all reduced to a DR_5_ of only 12%. What is relevant in screening is the risk of an event in a group compared with that of the whole population, which is achieved with the calculation of the detection rate for a specified false positive rate.

### Policy implications

Our findings are relevant to commercial providers of genetic tests and to researchers working on polygenic risk scores. Commercial providers could communicate individual test results to customers with greater clarity and relevance to performance in disease prediction; for example, by presenting the overlapping distributions of polygenic risk scores among those later affected and unaffected and by presenting an absolute measure of risk for an individual or group, which requires additional information on population average risk at a particular age over a specified time. At the same time, as already suggested,^42^ policy makers might wish to consider stricter regulation of commercial genetic tests based on polygenic risk scores, with a focus on clinical performance and not just assay performance (as indicated by the Royal Statistical Society Diagnostic Tests Working Group Report^43^), to protect the public from unrealistic expectations and already stretched public health systems from becoming overburdened by the management of false positive results. Researchers reporting studies on polygenic risk scores should present as a minimum: mean and standard deviation values for polygenic risk scores among later affected and unaffected individuals; overlap in their distributions; relevant performance metrics, such as the detection rate for a specified false positive rate (eg, DR_5_), avoiding the need to make this calculation indirectly^23^; and performance of polygenic risk scores with and without the inclusion of other variables so that users can judge the incremental benefit provided by the polygenic risk score itself.

Although our analysis showed the poor performance of polygenic risk scores in screening, prediction, and risk stratification, these scores might be useful in other situations. For example, polygenic scores might explain the variable penetrance of rare mutations in monogenic diseases (eg, hypertrophic cardiomyopathy or familial hypercholesterolaemia), and be used to help detect patients. Other predictive applications of genotyping also exist, for example in pharmacogenetic testing to optimise the efficacy and safety of medicines. Genotyping might also be of value in blood and tissue matching. Because genetic variation is transmitted from parents to offspring through a randomised process (like treatment allocation in a clinical trial), and is unaltered by disease, an important translational application arising from genomic discoveries could be providing evidence on disease causation and targets for pharmaceutical intervention.^44^

### Conclusion

Use of the appropriate metrics showed poor performance of polygenic risk scores in population screening, individual risk prediction, and population risk stratification. The wide scope and analytical approach of our study might help to resolve the debate on the value of polygenic risk scores, and avoid unjustified expectations about their role in the prediction and prevention of disease.

## Data Availability

Data are available in a public, open access repository. Data are available on the Polygenic Score Catalog website.
