# Supplementary material for: Performance of polygenic risk scores in screening, prediction, and risk stratification: secondary analysis of data in the Polygenic Score Catalog
Source: BMJ Med. 2023 Oct 17;2(1):e000554. doi: 10.1136/bmjmed-2023-000554 (PMC10582890; doi:10.1136/bmjmed-2023-000554)
Supplement: Supplementary data [file bmjmed-2023-000554supp002.pdf]

# Performance of polygenic risk scores in screening, prediction, and risk stratification – Extended Methods

**Hingorani AD., FMedSci[1,2,3,4], Gratton J., PhD[1, 2], Finan C., PhD[1,2,3,4], Schmidt AF., PhD[1,2,3,4,5], Patel R., PhD[1,2,3,4]., Sofat R., PhD[3,4,6], Kuan V., PhD[1,2,3], Langenberg C., PhD[7], Hemingway H., FMedSci[2,3,4,8], Morris JK., PhD[9], Wald NJ., FRS[8,9]**

[1] Institute of Cardiovascular Science, University College London, UK.

[2] UCL British Heart Foundation Research Accelerator.

[3] UCL National Institute of Health Research Biomedical Research Centre.

[4] Health Data Research, UK.

[5] University Medical Centre, Utrecht, Netherlands.

[6] Department of Pharmacology and Therapeutics, Institute of Systems, Molecular and Integrative Biology, University of Liverpool.

[7] Queen Mary University of London, UK.

[8] Institute of Health Informatics, University College London, UK.

[9] Population Health Research Institute, St. George's, University of London, UK.

Observed characteristics of polygenic scores are that they exhibit a Gaussian distribution, with the distribution being higher among those affected than among those unaffected, with the same $SD$ in both groups. Consequently, the proportional difference in disease risk is the same for any given absolute difference in polygenic score value, from any starting level (i.e., a log-linear relationship). These properties enable calculation of metrics useful in the evaluation of performance is screening, individual risk prediction and population risk stratification.

*Deriving $DR_5$ from $HR$ or $OR$ per-SD*

The detection rate ($DR$) for a given false positive rate can be derived from the $OR$ or $HR$ per-$SD$. The $HR$ is the relative risk of the event occurring at any point in time compared to a control group of people and therefore all the assumptions for the $OR$ are also applicable for the $HR$. Consider the Gaussian frequency distributions of polygenic risk scores among those unaffected ($U$) and those who become affected by disease ($A$). The mean polygenic score value among those unaffected is $\mu_U$ and among those affected is $\mu_A$, and both distributions have the same $SD$ ($\sigma$)

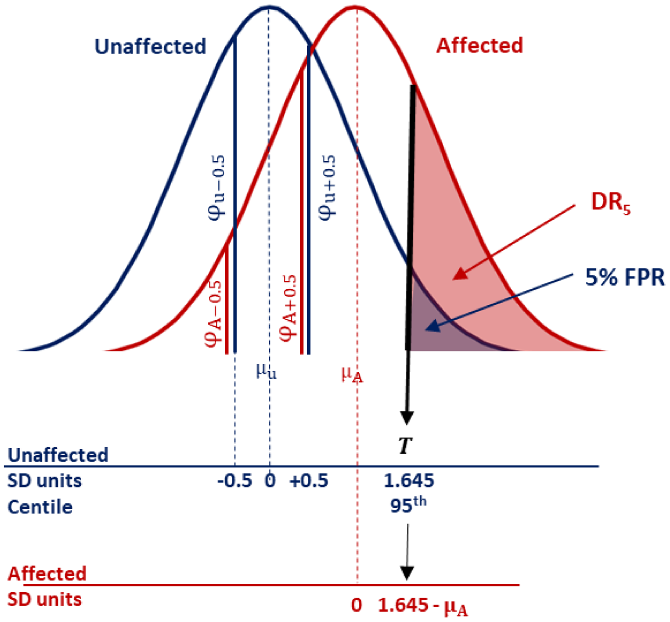


The $OR$ per-$SD$ can be considered to be equal to the ratios of the probability density function values $\varphi$, for affected ($\varphi_A$) vs. unaffected individuals ($\varphi_U$) at Z-score values for the distribution among the unaffected (i.e. $Z_U$ values) that are 1-$SD$ apart, for example

corresponding to values at $+0.5\ SD$ and $-0.5SD$. In the figure, the values are shown by vertical red and blue, lines respectively. Thus,

$$OR_{SD} = \frac{\left({}^{\varphi_A}/_{\varphi_U}\right)_{+0.5}}{\left({}^{\varphi_A}/_{\varphi_U}\right)_{-0.5}}$$

Where $x$ is the value of the variate, the probability density function $\varphi$ is given by:

$$\varphi(\mu, \sigma, x) = \frac{1}{\sigma\sqrt{2\pi}} e^{-\frac{1}{2}\left(\frac{x-\mu}{\sigma}\right)^2}$$

For the distribution in the unaffected, $\mu$ = 0, $\sigma$ =1

$$\varphi U(0,1,x) = \frac{1}{1\sqrt{2\pi}} e^{-\frac{1}{2}\left(\frac{x}{1}\right)^2}$$

$$\varphi U(0,1,-0.5) = \frac{1}{1\sqrt{2\pi}} e^{-\frac{1}{2}\left(\frac{-0.5}{1}\right)^2}$$

$$\varphi U(0,1,+0.5) = \frac{1}{1\sqrt{2\pi}} e^{-\frac{1}{2}\left(\frac{+0.5}{1}\right)^2}$$

Since $\varphi_{U+0.5}$= $\varphi_{U-0.5}$,

$$OR_{SD} = \frac{(\varphi_{A+0.5})}{(\varphi_{A-0.5})}$$

For the distribution in the affected, $\mu$ = $\mu_A$, $\sigma$ =1

$$\varphi A(\mu_A, \sigma, -0.5) = \frac{1}{1\sqrt{2\pi}} e^{-\frac{1}{2}\left(\frac{-\mu_A-0.5}{1}\right)^2}$$

$$\varphi A(\mu_A, \sigma, +0.5) = \frac{1}{1\sqrt{2\pi}} e^{-\frac{1}{2}\left(\frac{0.5-\mu_A}{1}\right)^2}$$

Thus,

$$OR_{SD} = \frac{\frac{1}{1\sqrt{2\pi}} e^{-\frac{1}{2}\left(\frac{0.5-\mu_A}{1}\right)^2}}{\frac{1}{1\sqrt{2\pi}} e^{-\frac{1}{2}\left(\frac{-\mu_A-0.5}{1}\right)^2}}$$

Cancelling $\frac{1}{1\sqrt{2\pi}}$,

$$OR_{SD} = \frac{e^{-\frac{1}{2}(0.5-\mu_A)^2}}{e^{-\frac{1}{2}(-\mu_A-0.5\,)^2}}$$

$$OR_{SD} = e^{[-\frac{1}{2}(0.5-\mu_A)^2+\frac{1}{2}(-\mu_A-0.5\,)^2]}$$

$$OR_{SD} = e^{-\frac{1}{2}[\,(0.5-\mu_A)^2-\,(-\mu_A-0.5\,)^2]}$$

$$OR_{SD} = e^{-\frac{1}{2}[-2\mu_A]}$$

$$OR_{SD} = e^{\mu_A}$$

$$\mu_A = \ln OR_{SD}$$

Also from the figure,

$$DR_5 = 1 - \emptyset(1.645 - \mu_A)$$

where Ø corresponds to the cumulative distribution function ($CDF$) corresponding to the area under the normal distribution among affecteds at a $Z$-value of $1.645 - \mu_A$. (The $1.645$ is the $95^{\text{th}}$ centile of the distribution among unaffecteds, which is the threshold for a $5\%$ false positive rate). Because of the symmetry of the normal distribution,

$$DR_5 = \emptyset(\mu_A - 1.645)$$

Thus, from equation 2,

$$DR_5 = \emptyset(\ln OR_{SD} - 1.645)$$

Consider, as an example, an $OR_{SD} = 5$.

In this example, $\ln OR_{SD} = \mu_A = 1.609$.

Thus, from equation 3,

$$DR_5 = \emptyset(1.609 - 1.645)$$

$$DR_5 = \emptyset(-0.03556)$$

$$DR_5 = 0.485816$$

Thus an $OR$ per-$SD$ of $5$ corresponds to a $49\%$ detection rate for a $5\%$ false positive rate.

*Estimating $DR_5$ from AUC and C-index*

The following derivation is adopted from Wald and Bestwick (Ref 23).

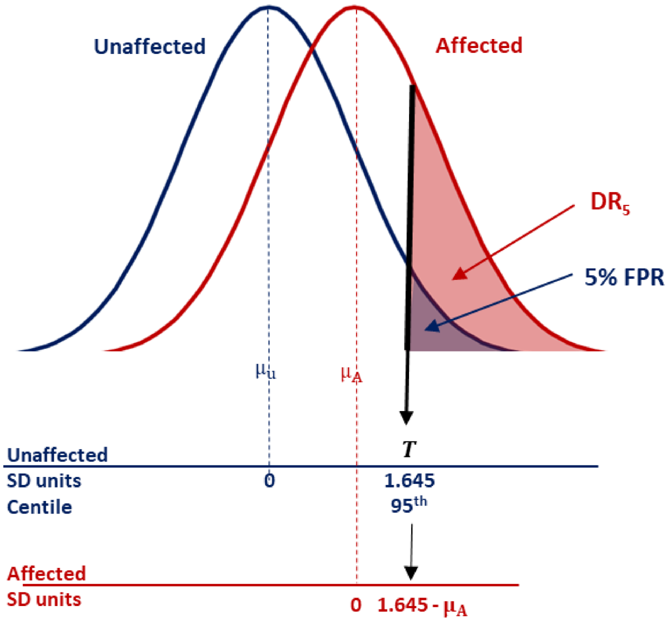


The $DR$ at a cut-point value $T$, is given by,

$$DR = 1 - \emptyset\left(\frac{T - \mu_A}{\sigma_A}\right)$$

Because of the symmetry of the normal distribution,

$$DR = \emptyset\left(\frac{\mu_A - T}{\sigma_A}\right)$$

By the same argument,

$$FPR = \emptyset\left(\frac{\mu_U - T}{\sigma_U}\right)$$

The $Z$-score corresponding to the $DR$ expressed in terms of the affected distribution is:

$$\emptyset^{-1}(DR) = \frac{\mu_A - T}{\sigma_A}$$

The $Z$-score corresponding to the FPR expressed in terms of the unaffected distribution is:

$$\emptyset^{-1}(FPR) = \frac{\mu_U - T}{\sigma_U}$$

Writing both equations in terms of $T$

$$T = \mu_A - \sigma_A \emptyset^{-1}(DR)$$

$$T = \mu_U - \sigma_U \emptyset^{-1}(FPR)$$

Thus,

$$\mu_A - \sigma_A \emptyset^{-1}(DR) = \mu_U - \sigma_U \emptyset^{-1}(FPR)$$

Making $DR$ the subject,

$$\sigma_A \emptyset^{-1}(DR) = \mu_A - \mu_U + \sigma_U \emptyset^{-1}(FPR)$$

$$\emptyset^{-1}(DR) = \frac{\mu_A - \mu_U + \sigma_U \emptyset^{-1}(FPR)}{\sigma_A}$$

$$DR = \emptyset\left(\frac{\mu_A - \mu_U}{\sigma_A} + \frac{\sigma_U \emptyset^{-1}(FPR)}{\sigma_A}\right)$$

Considering the standard normal distribution and assuming equal standard deviation in unaffected and affected groups, and $\mu_U = 0$.

$$DR = \emptyset\left(\frac{\mu_A}{1} + \frac{1 \cdot \emptyset^{-1}(FPR)}{1}\right)$$

$$DR = \emptyset\left(\mu_A + \emptyset^{-1}(FPR)\right)$$

The area under the ROC curve (*AUC*), or equivalently the $C$-index, is the probability that an affected individual drawn at random $(A)$ has a higher polygenic risk score than an unaffected individual drawn at random $(U)$ i.e.

$$P(A > U) = P(A - U > 0)$$

The $AUC$ is the therefore the CDF for the distribution of differences (the variances sum). Thus,

$$AUC = \emptyset\left(\frac{\mu_A - \mu_U}{\sqrt{\sigma_A^2 + \sigma_U^2}}\right)$$

$$\emptyset^{-1}(AUC) = \frac{\mu_A - \mu_U}{\sqrt{\sigma_A^2 + \sigma_U^2}}$$

Given that for the standard normal distribution $\mu_U = 0$ and if we assume equal standard deviation for the distributions for affected and unaffected individuals, $\sigma_U = \sigma_A = 1$,

$$\emptyset^{-1}(AUC) = \frac{\mu_A}{\sqrt{2}}$$

For example, if the $AUC$ is $0.7$,

$$\emptyset^{-1}(0.7) = 0.524$$

Thus,

$$\frac{\mu_A}{\sqrt{2}} = 0.524$$

$$\mu_A = 0.524 \cdot \sqrt{2}$$

$$\mu_A = 0.742$$

Since,

$$DR = \emptyset\left(\mu_A + \emptyset^{-1}(FPR)\right)$$

the $DR_5$ corresponding to an $AUC$ of $0.7$ is,

$$DR_5 = \emptyset\left(0.742 + \emptyset^{-1}(0.05)\right)$$

$$DR_5 = \emptyset(0.742 + (-1.645)$$

$$DR_5 = \emptyset(-0.903)$$

$$DR_5 = 0.183$$

Thus an $AUC$ of $0.7$ corresponds to a detection rate of $18\%$ for a $5\%$ false positive rate.

*Calculating the likelihood ratio and odds of becoming affected given a positive test result ($OAPR$)*

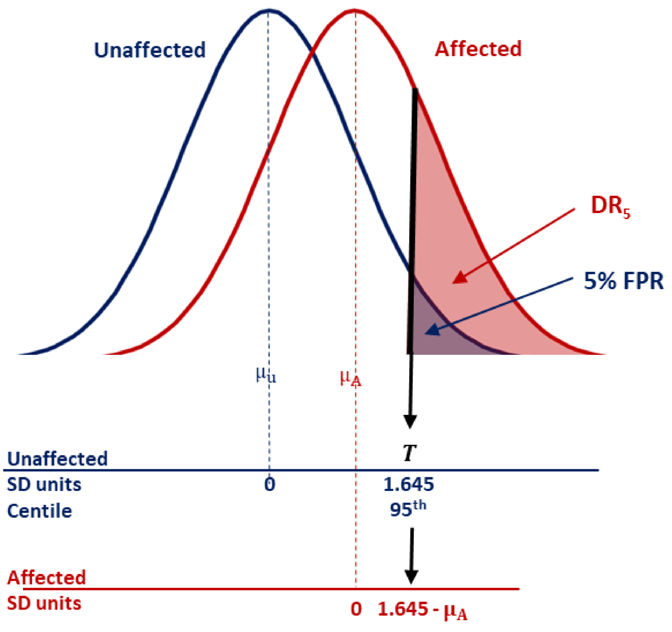


In evaluating the performance of a polygenic risk score as a screening test, we calculated the likelihood ratio for a positive result (i.e., a polygenic risk score at or above a pre-specified

cut-off) as the ratio $DR/FPR$. The likelihood ratio for a test cut-off with a $5\%$ $FPR$ is given by $^{DR_5}/_{5\%}$, where $DR_5$ is expressed as a percentage. The $OAPR$ is calculated by multiplying the background odds of disease by the likelihood ratio for a positive test result. For example, if the background odds of disease in the population is $1:9$ and the $DR_5$ is $15\%$, the likelihood ratio is $15/5 = 3$ and the $OAPR = 3:9$ or $1:3$.

*Calculating the likelihood ratio and odds of becoming affected for an individual with a given polygenic score result*

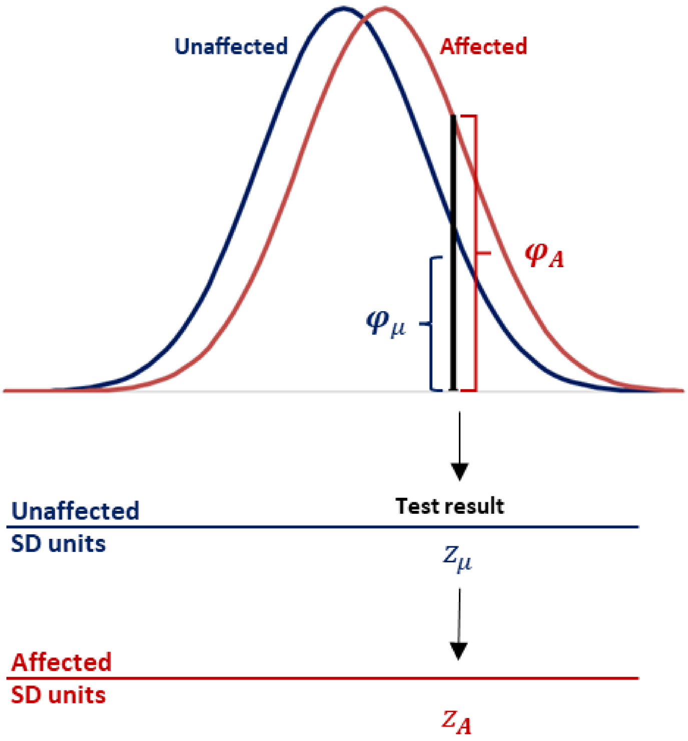


To compute how many times more likely a given polygenic score result is to arise from an affected than unaffected individual, we compared the heights of the standard distribution curves for affected and unaffected individuals at that value (the likelihood ratio; $LR$). In the illustration shown, this is given by the ratio $^{\varphi_A}/_{\varphi_U}$.

$$LR = {}^{\varphi_A}/_{\varphi_U}$$

These heights can be calculated using the equation for the Gaussian distribution:

$$LR = \frac{\frac{1}{\sigma\sqrt{2\pi}} e^{-\frac{1}{2}(Z_A)^2}}{\frac{1}{\sigma\sqrt{2\pi}} e^{-\frac{1}{2}(Z_U)^2}}$$

For example, for a polygenic risk score with a performance metric expressed as an $OR_{SD} = 1.61$:

$$\mu_A = \ln OR_{SD} = 0.48$$

A polygenic score at the 75th centile of the distribution for unaffected individuals yields,

$Z_U = 0.67; \varphi_U = 0.32$

$Z_A = 0.2, \varphi_A = 0.39$

$LR = {}^{\varphi_A}/_{\varphi_U} = {}^{0.39}/_{0.32} = 1.23$

If the background odds of disease in the population is $1:9$, an individual whose polygenic score is at the 75th centile of the distribution among unaffecteds has an odds of being affected of $(1.23 \times 1):9 \approx 1:7$.

*Calculating the likelihood ratio and odds of becoming affected for a particular polygenic risk score group*

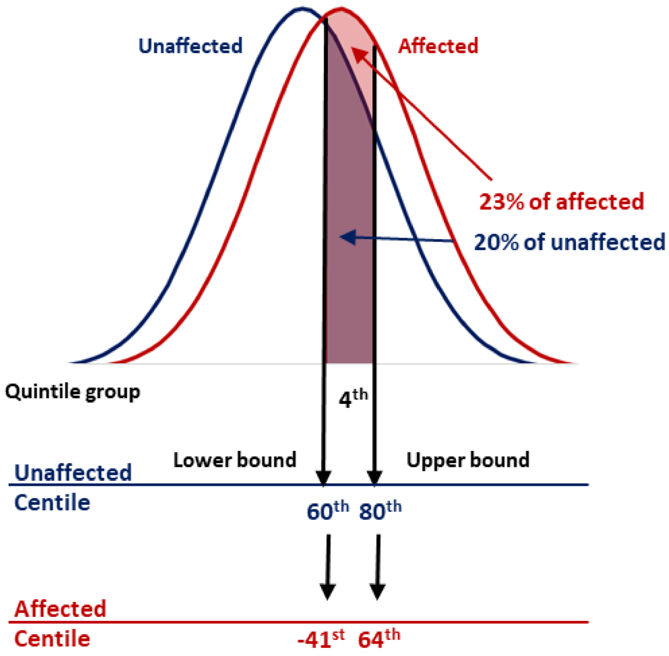


In evaluating polygenic risk scores in risk stratification, we calculated the $LR$ as the ratio of areas under the distributions for affected and unaffected individuals in each polygenic score quantile (e.g., each fifth of the polygenic score distribution with respect to the unaffected, as shown in the figure). We then multiplied the background odds of disease by the corresponding likelihood ratio to determine the odds of becoming affected for each quantile of the distribution. For example, for individuals in the fourth quintile, if the $LR$ is $1.2$ (figure) and the background odds of disease in the population is $1:9$, the odds of becoming affected for this group is $1.2:9 \approx 1:8$.
